# Coinfection of paracoccidioidomycosis and mucocutaneous leishmaniasis: a case report and systematic review

**DOI:** 10.3389/fmed.2026.1892049

**Published:** 2026-10-08

**Authors:** Paula Valentina Bayona, Iván Salcedo, Luis Alejandro Charry, Brayan Bayona, Tomás Martín, Luisa Fernanda Murcia, Paula Ximena Pavia

**Affiliations:** 1 Department of Medical Clinics, Hospital Universitario Mayor Méderi, Universidad del Rosario, Bogotá, Colombia; 2 Department of Internal Medicine, Fundación Cardioinfantil, Bogotá, Colombia; 3 Department of Medicine, Division of Health Sciences, Universidad del Norte, Barranquilla, Colombia; 4 ADOM Salud IPS, Bogotá, Colombia; 5 Research Department, Hospital Universitario Mayor Méderi, Universidad del Rosario, Bogotá, Colombia; 6 Infectious Diseases and Tropical Medicine Institute, Hospital Universitario Mayor Méderi, Universidad del Rosario, Bogotá D.C, Colombia

**Keywords:** coinfection, granulomatous paracoccidioidomycosis, pulmonary paracoccidioidomycosis, mucocutaneous leishmaniasis, neglected tropical diseases

## Abstract

**Background:**

Systemic mycoses and neglected tropical diseases, such as paracoccidioidomycosis (PCM) and leishmaniasis, remain major public health concerns in endemic regions of Latin America. Their coinfection is exceptionally rare and scarcely reported although these infections share overlapping ecological niches and risk factors across multiple endemic countries, including Colombia.

**Methods:**

We report the first documented case of coinfection by PCM and mucocutaneous leishmaniasis in an elderly male agricultural worker from a tropical endemic region in Colombia. The patient presented with upper gastrointestinal bleeding, chronic respiratory symptoms, long-standing nasal and oral mucocutaneous lesions, and severe malnutrition. A systematic literature review was also conducted following PRISMA guidelines to identify all previously reported cases of PCM–leishmaniasis coinfection up to April 2026. The review protocol was registered in Open Science Framework (https://doi.org/10.17605/OSF.IO/9T5GM).

**Results:**

Histopathological examination established pulmonary PCM, and additional nasal and oral lesions biopsies confirmed mucocutaneous leishmaniasis with granulomatous PCM involvement. The systematic review identified 381 records, from which only two previously published cases were eligible; three cases were analyzed together with the index case. All patients were adult males from endemic areas with relevant environmental exposure and no evidence of a recognized immunosuppressive disorder or immunosuppressive therapy. The clinical manifestations were highly heterogeneous, ranging from localized mucocutaneous involvement to disseminated disease with pulmonary and laryngeal compromise. Notably, the Colombian case expanded the known clinical spectrum by including upper gastrointestinal bleeding and severe malnutrition. In all cases, the diagnosis relied on histopathological confirmation, and treatment with amphotericin B followed by itraconazole resulted in favorable clinical outcomes.

**Conclusion:**

PCM–leishmaniasis coinfection is a rare but clinically significant entity that poses important diagnostic challenges, particularly in endemic settings. This case highlights the diagnostic challenges posed by overlapping clinical presentations and underscores the importance of maintaining a high index of suspicion and performing comprehensive diagnostic evaluations in patients from regions where both infections may coexist.

## 1 Introduction

PCM is a multisystem infectious disease characterized by a chronic and progressive course, with an endemic distribution in Latin America (1). The etiological agent is *Paracoccidioides* spp. a species complex comprising seven described species, with *P. brasiliensis* being the first identified and the most extensively studied in clinical and epidemiological settings (2). The diagnosis is based on epidemiological, clinical, and laboratory criteria. A proven case corresponds to a patient with a compatible clinical presentation and microbiological confirmation through the identification of *Paracoccidioides* spp. by direct microscopic examination and/or fungus isolation in culture (3). *Paracoccidioides* spp. is generally susceptible to most azole antifungals, with amphotericin B reserved for severe cases. Long-term sulfonamide treatment is recommended after the acute phase to prevent relapses (4, 5).

From an epidemiological perspective, PCM is restricted to Latin America, ranging from Mexico to Argentina, with Brazil accounting for most cases. Cases have been described in nine departments in Colombia, with *Paracoccidioides restrepiensis* identified as the most frequent species (5, 6). The limited availability of epidemiological data over the past two decades, together with forced displacement and migration phenomena, may have altered the eco-epidemiological behavior of PCM. Additionally, since PCM is not a notifiable disease, reliable estimates of its incidence and prevalence are lacking, and it is therefore classified as a neglected tropical disease (NTD) (7).

Similarly, leishmaniasis is a chronic infectious NTD caused by flagellated protozoa of the genus *Leishmania* spp. transmitted as a zoonosis through the bite of infected female sand flies of the genus *Lutzomyia* spp. (8). More than 20 species infect humans, causing three main clinical forms: cutaneous, mucocutaneous, and visceral, with the latter being associated with greater severity, especially in immunocompromised individuals (8, 9). The diagnosis of cutaneous leishmaniasis relies on epidemiological exposure, clinical suspicion, and parasitological confirmation through direct microscopy, histopathology, and, when indicated, culture. Treatment in Colombia is mainly based on pentavalent antimonials, such as meglumine antimoniate, with alternative therapies reserved for selected cases (10).

Colombia is endemic for leishmaniasis throughout most of its territory, except San Andrés, Providencia, and Bogotá D.C., which primarily affects rural populations due to multiple environmental and socioeconomic risk factors. According to surveillance data from 2025, cutaneous leishmaniasis remains the most prevalent form, whereas mucosal and visceral manifestations occur less frequently (11).

Given that PCM and leishmaniasis are neglected tropical diseases of public health relevance in Colombia, their unusual presentations pose significant diagnostic and therapeutic challenges. In this context, we present the first reported case in the Colombian literature of coinfection with *Paracoccidioides* spp. and *Leishmania* spp. accompanied by a systematic literature review, highlighting the need for a high index of diagnostic suspicion in endemic areas.

## 2 Case report

A 61-year-old man from a rural region of the Vichada department in Colombia, who worked as a farmer and wood-based charcoal producer was referred to a tertiary-care hospital in Bogotá after presenting with hemorrhagic shock. The patient required blood transfusions and vasopressor support due to upper gastrointestinal bleeding caused by a Forrest III duodenal ulcer associated with *Helicobacter pylori* infection.

During his hospitalization, the patient developed hypoxemia requiring supplemental oxygen. Chest imaging revealed a nodule with a reverse halo sign, along with additional solid nodules with spiculated margins and peribronchovascular interstitial thickening, suggesting an underlying infectious process (Figure 1). Given these findings, the Pulmonology team performed flexible bronchoscopy with transbronchial biopsy.

**Figure 1:**
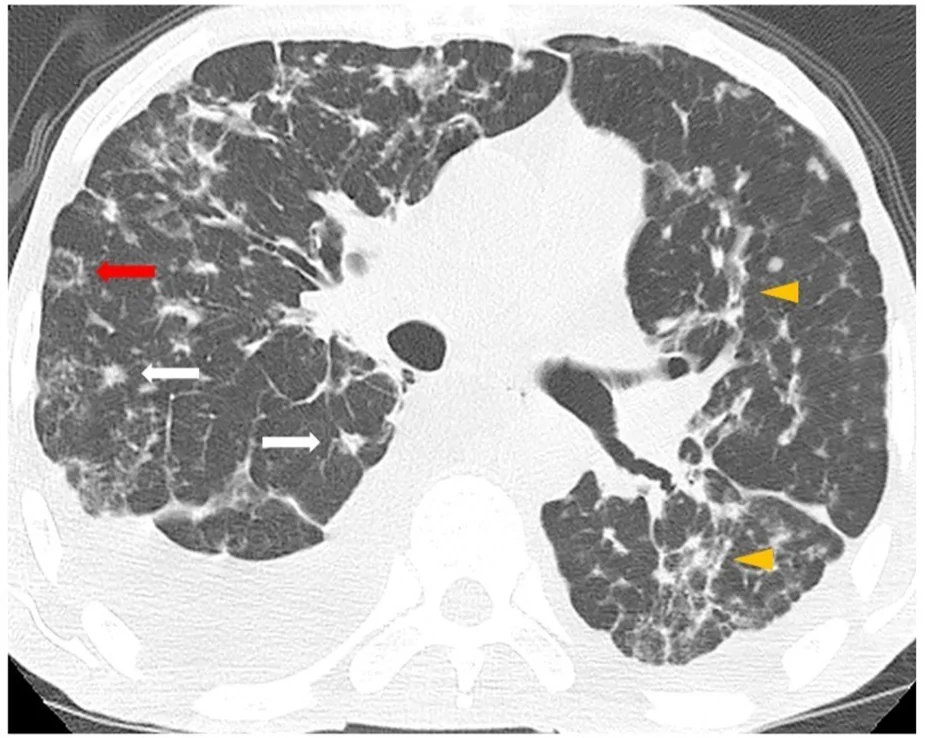
Chest computed tomography showing a nodule with a reverse halo sign (red arrow). Additional findings include spiculated margins (white arrow) and peribronchovascular interstitial thickening (yellow arrowhead).

The initial microscopic examination of the bronchoalveolar lavage specimen using a potassium hydroxide (KOH) preparation demonstrated yeast-like structures reported as blastoconidia. Fungal cultures performed on the same specimen yielded only *Candida albicans*, with no isolation of *Paracoccidioides* spp. Urine Histoplasma antigen testing using an enzyme immunoassay (Clarus Histoplasma GM EIA, IMMY Inc., Norman, OK, USA) was positive (31.6 ng/mL), leading to a presumptive diagnosis of disseminated histoplasmosis. However, subsequent histopathological analysis of the transbronchial biopsy demonstrated multiple yeast-like fungal structures, including budding forms composed of a mother cell and a peripheral daughter bud, morphologically compatible with *Paracoccidioides* spp. supporting the diagnosis of PCM (Figure 2)

**Figure 2:**
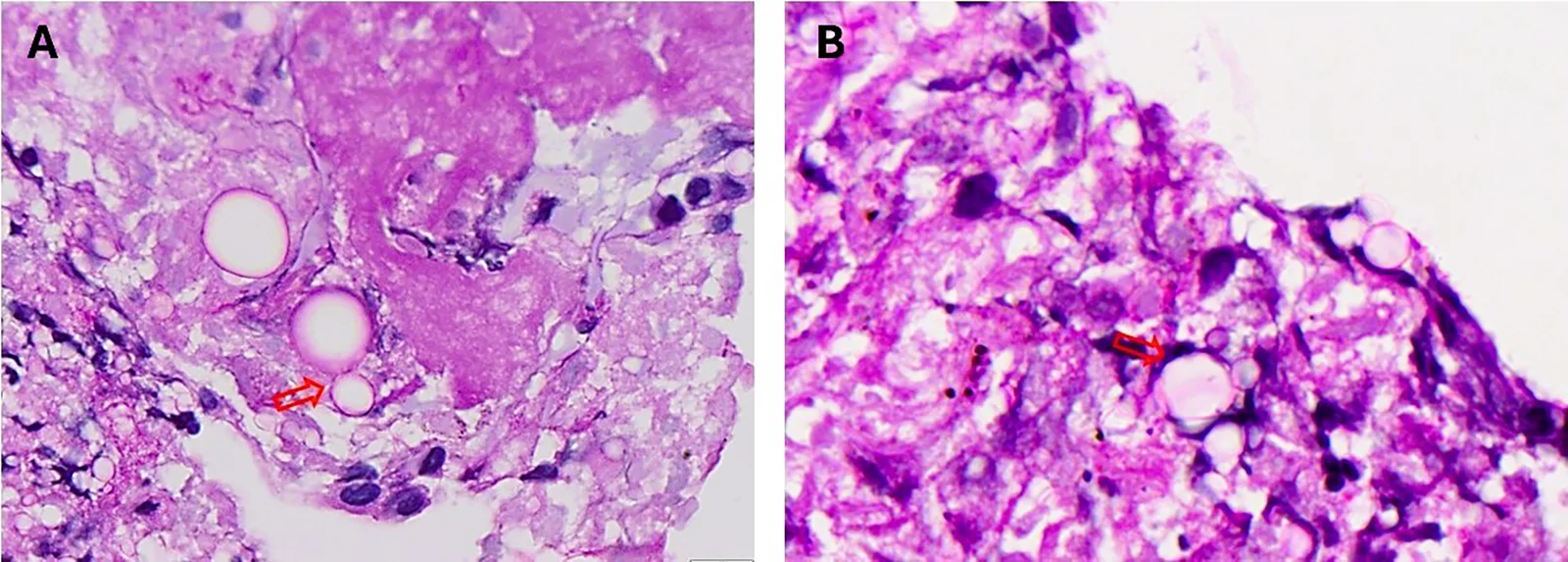
Histopathological findings in transbronchial lung biopsy (TBL), periodic acid-Schiff (PAS) stain (1000×). **(A)** Multiple round yeast-like structures with thick refractile walls are identified within the pulmonary parenchyma. Several fungal elements exhibit features suggestive of budding, supporting their interpretation as structures morphologically compatible with *Paracoccidioides* spp. **(B)** Representative budding yeast composed of a large mother cell associated with a smaller peripheral daughter bud (arrow), resulting in a “Mickey Mouse”-like configuration compatible with *Paracoccidioides* spp.

The patient's occupational exposure, tobacco use, and chronic alcohol consumption represented recognized risk factors for chronic PCM. He also presented with severe malnutrition, reflecting a compromised overall health status at the time of diagnosis.

In addition, the patient presented with ulcerative and erosive mucocutaneous lesions that had been progressively evolving for approximately 6 months, involving the nasal columella, the edentulous alveolar ridge at the level of teeth 21–22, and the hard palate.

Histopathologic evaluation of the biopsy obtained from the nasal septal lesion demonstrated granulomatous inflammation containing structures morphologically consistent with *Leishmania* amastigotes, confirming mucocutaneous leishmaniasis (Figure 3). Otorhinolaryngological examination revealed an ulcerative lesion involving the nasal septum and adjacent mucocutaneous tissues, from which the diagnostic biopsy was obtained. In contrast, the palatal and alveolar lesions showed chronic and acute necrotizing granulomatous inflammation associated with systemic paracoccidioidomycosis.

**Figure 3:**
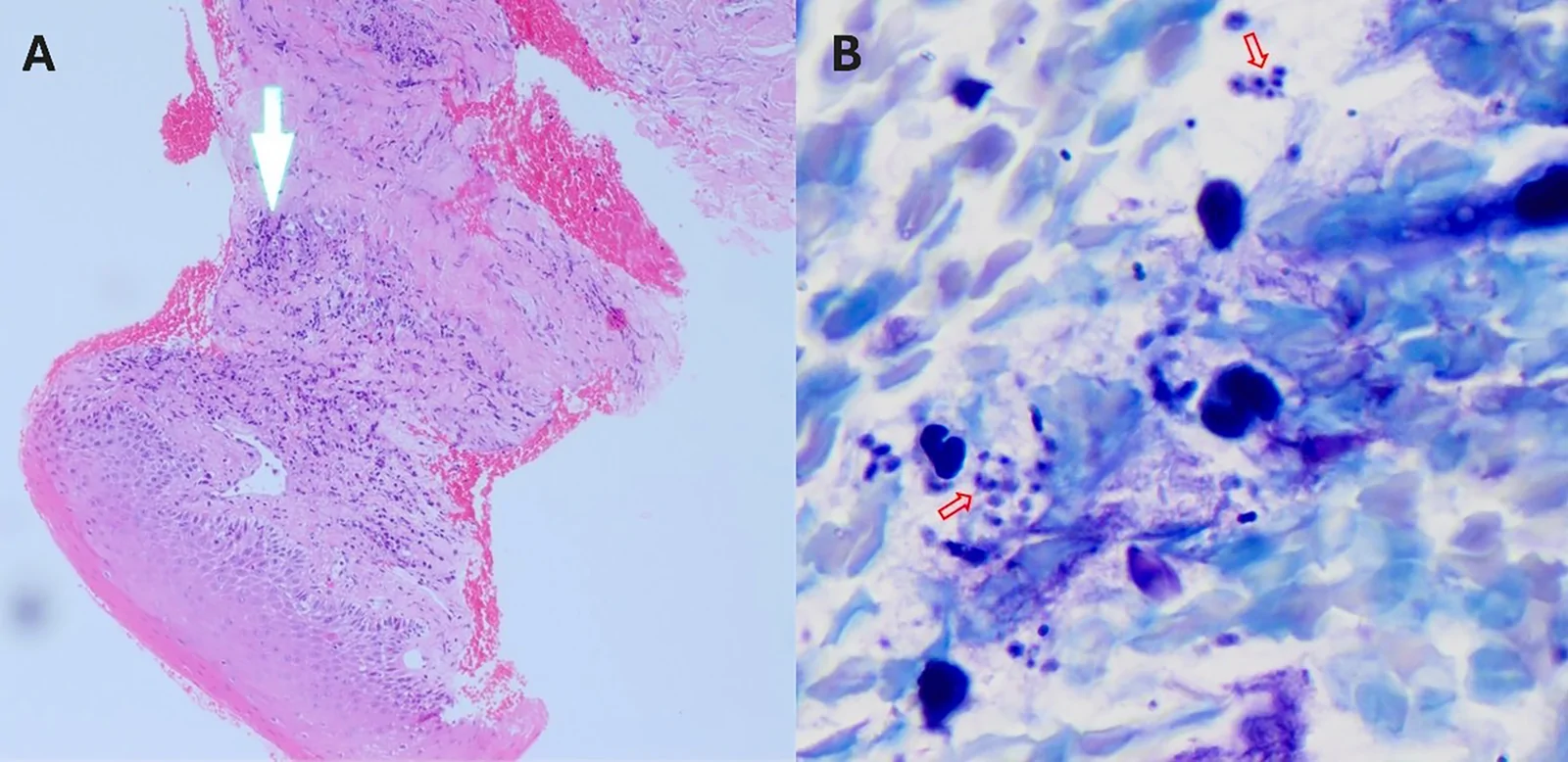
Histopathological findings of a nasal mucocutaneous lesion consistent with leishmaniasis **(A)** hematoxylin and eosin (H&E) stain (40×) showing a chronic dermal inflammatory infiltrate with histiocytic predominance (arrow). **(B)** Histopathological examination of the mucocutaneous lesion (Giemsa stain, 1000×) demonstrating a large mononuclear cell containing multiple clustered basophilic intracellular structures and morphologically compatible with *Leishmania* amastigotes (red arrows).

A final diagnosis of systemic paracoccidioidomycosis with concomitant mucocutaneous leishmaniasis was established based on the integration of clinical presentation, diagnostic imaging, and histopathologic results. Additional serological and molecular diagnostic methods were not performed because the histopathological findings provided definitive diagnostic evidence and these tests were not routinely available in our institution.

During hospitalization, pulmonary findings, including a cavitated nodule in the middle lobe, consolidation in the left lower lobe, and right pleural effusion, complicated the clinical course (Figure 4). The patient also developed a spontaneous left-sided pneumothorax that subsequently progressed to a multiloculated pneumothorax. Therefore, the thoracic surgery team evaluated the patient, who performed a closed thoracostomy with satisfactory resolution of the condition. In addition, he developed a bloodstream infection caused by *Pantoea agglomerans*, which resolved following treatment with carbapenem antibiotics.

**Figure 4:**
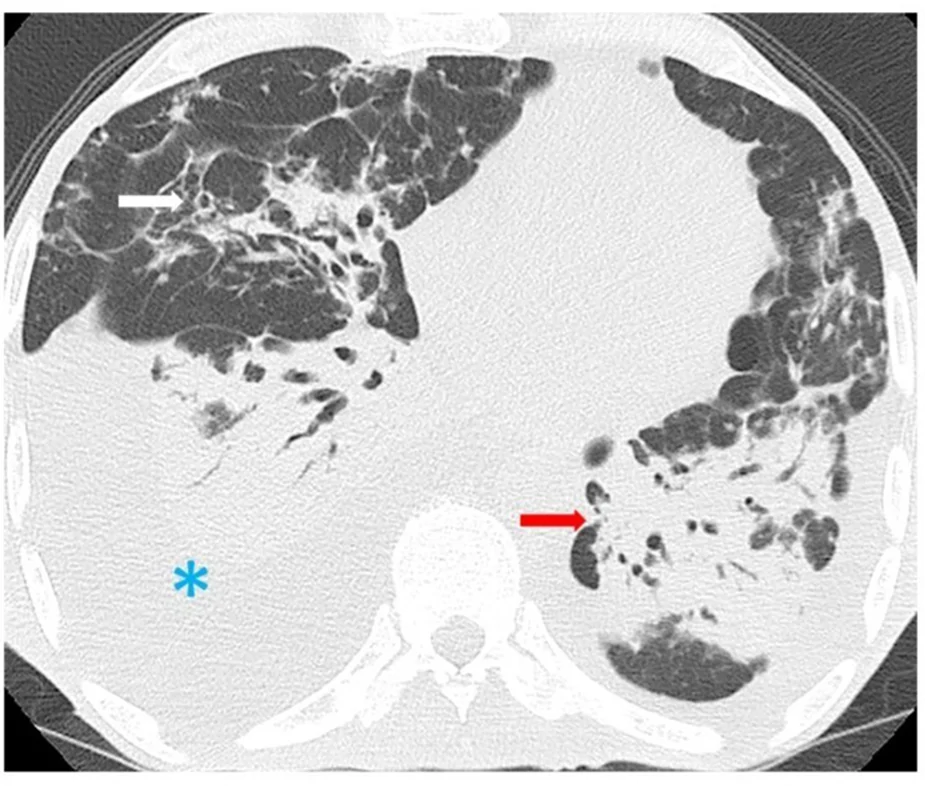
Chest computed tomography showing a cavitated nodule in the middle lobe (white arrow) and consolidation in the left lower lobe (red arrow). Free right pleural effusion is also observed (asterisk).

A comprehensive diagnostic evaluation ruled out infection with human immunodeficiency virus (HIV), *Treponema pallidum*, and *Mycobacterium tuberculosis*. Age-appropriate cancer screening revealed no evidence of malignancy. Autoimmune conditions with overlapping clinical features, including antineutrophil cytoplasmic antibody (ANCA)-associated vasculitides, were also excluded from the study. Baseline laboratory testing showed a normal leukocyte count (7.76 × 10^3^ cells/mm^3^), relative lymphopenia (9.9%), eosinopenia (0.3%), preserved glycemic status (HbA1c 5.6%), preserved renal function (creatinine 0.60 mg/dL), and preserved hepatic function (AST 14.48 U/L, ALT 10.10 U/L, total bilirubin 0.40 mg/dL). The patient denied chronic medication use, including corticosteroids or other immunosuppressive agents. However, severe hypoalbuminemia (2.11 g/dL), consistent with malnutrition, together with chronic alcohol consumption, active systemic infection, and subsequent nosocomial bloodstream infection, may have affected host defense mechanisms. Therefore, although no underlying immunosuppressive condition was recognized, the patient's immune status could not be fully characterized.

The most relevant phases of the patient’s clinical course, including symptom onset, diagnostic evaluation, and treatment, are summarized in a timeline to facilitate the interpretation of disease progression and clinical decision-making (Figure 5).

**Figure 5:**
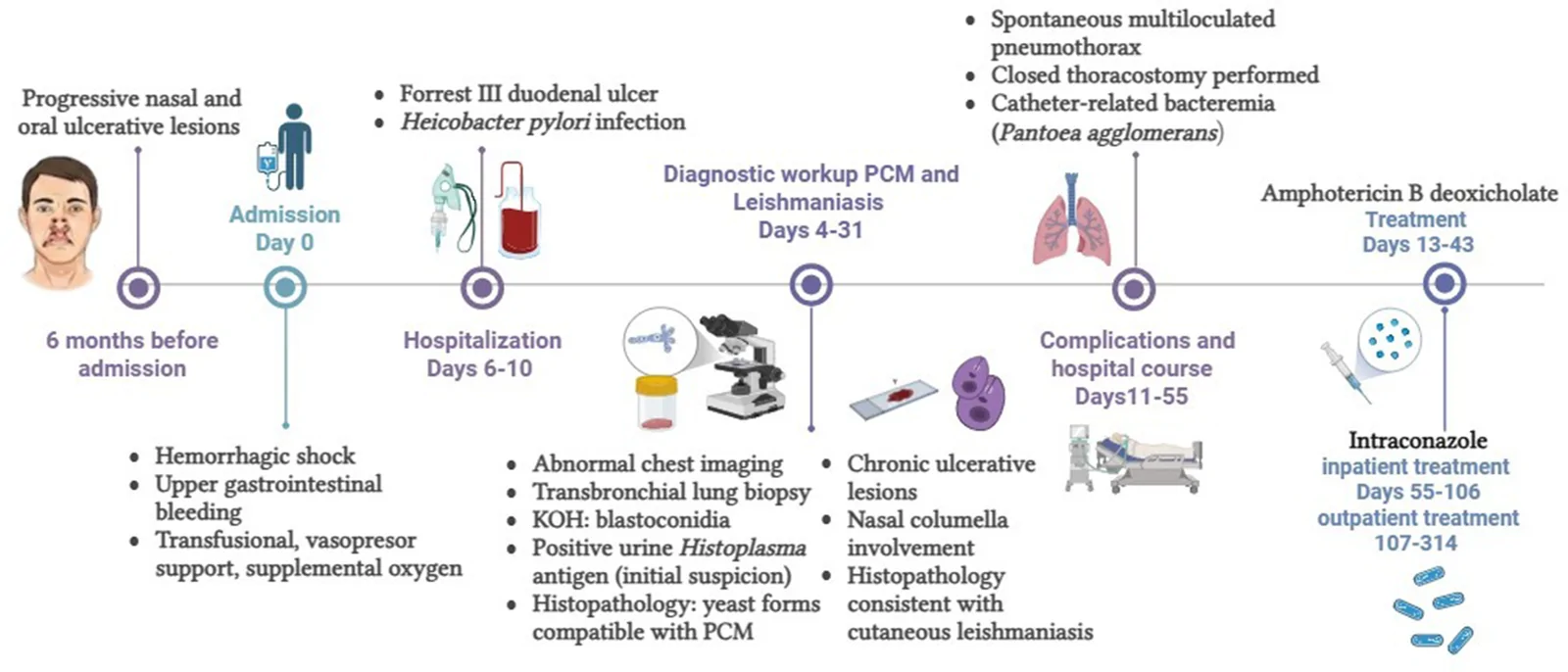
Clinical timeline summarizing hospitalization, diagnostic workup, treatment, and major complications related to paracoccidioidomycosis and mucocutaneous leishmaniasis. Time points are presented as relative clinical phases.

### 2.1 Treatment and follow-up

Amphotericin B deoxycholate was initiated empirically because of suspected disseminated histoplasmosis based on a positive urinary *Histoplasma* antigen test. Following histopathologic confirmation of PCM and identification of *Leishmania* spp. in the nasal septum biopsy, amphotericin B was continued because of its activity against both pathogens. Although a specific antileishmanial regimen was considered, it was not added to avoid additional toxicity.

Amphotericin B deoxycholate was administered at 1 mg/kg/day in two 15-day cycles, reaching a cumulative dose of 30 mg/kg. Renal function remained within normal limits throughout treatment. The main adverse effects were hypokalemia and hypomagnesemia, which were managed with electrolyte supplementation. No infusion-related reactions or other significant adverse events were observed.

After completion of amphotericin B therapy, itraconazole 200 mg/day was initiated as maintenance therapy for PCM, with a planned duration of one year. Therapeutic drug monitoring was not performed because it is not routinely available at our institution. Liver function tests were normal before itraconazole initiation, although complete biochemical monitoring during maintenance therapy was not available.

Treatment response was assessed separately for each infection. The nasal lesion showed progressive clinical improvement with mucosal healing, while pulmonary PCM demonstrated radiological improvement on follow-up chest CT performed after amphotericin B therapy. Itraconazole maintenance therapy was continued for 12 months; however, no follow-up imaging was available during the outpatient treatment period. The patient was discharged on itraconazole and reported adherence during the first four months of follow-up; however, he was subsequently lost to follow-up after returning to his rural place of residence. Therefore, despite documented clinical and radiological improvement, the available follow-up is insufficient to exclude late relapse.

## 3 Systematic review of literature

### 3.1 Methods

This systematic review was conducted following the JBI Manual for Evidence Synthesis (12) and the PRISMA guidelines (13).

### 3.2 Review question

The review was guided by the following research question: What are the clinical, diagnostic, therapeutic, and epidemiological characteristics of reported cases of coinfection with *Paracoccidioides* spp. and *Leishmania* spp. The review question was developed using the Population–Concept–Context (PCC) framework, as summarized below in the Table 1.

**Table 1:** Population–concept–context framework.

Framework component	Description
Population	Human subjects with laboratory-confirmed coinfection with *Paracoccidioides* spp. and *Leishmania* spp. with no restrictions on age, sex, or geographic location.
Condition	Coinfection defined as microbiological, histopathological, or molecular confirmation of both *Paracoccidioides* spp. and *Leishmania* spp. in the same patient, with evidence of concurrent active infection during the same clinical episode. Reports describing previous or resolved infection, residual sequelae without evidence of active disease, or cases in which only one pathogen was laboratory confirmed were excluded.
Outcomes of interest	Epidemiological characteristics, clinical manifestations, diagnostic methods, treatment regimens, and clinical outcomes (resolution, relapse, or mortality).

#### 3.1.1 Data sources and searches

A comprehensive search was performed across MEDLINE (Ovid), Embase (Ovid), Scopus, LILACS, and Epistemonikos from inception to April 14 2026. The search strategy combined controlled vocabulary with free-text terms, synonyms, spelling variants, and Boolean operators related to paracoccidioidomycosis, leishmaniasi*s*, and coinfection, and was adapted to the syntax of each database. No language restrictions were applied. Gray literature sources and the reference lists of all included studies were manually screened to identify additional eligible reports. The complete search strategies for all databases are provided in Supplementary Table S1.

#### 3.1.2 Eligibility criteria and selection criteria

We included case reports of human subjects with laboratory-confirmed coinfection, defined as microbiological, histopathological, or molecular confirmation of both *Paracoccidioides* spp. and *Leishmania* spp. in the same patient, with evidence of concurrent active infection during the same clinical episode. Reports describing previous or resolved infection, residual sequelae without evidence of active disease, or cases in which only one pathogen was laboratory confirmed were excluded. No restrictions were applied regarding age, sex, or geographic location. Conference abstracts, posters, and other publications lacking sufficient methodological and clinical information for eligibility assessment, data extraction, and quality appraisal were excluded. Retrieved records were managed using Rayyan (14), and duplicates were removed. Two reviewers independently screened the titles and abstracts, followed by a full-text assessment of the potentially eligible studies. Disagreements were resolved through consensus, and the selection process was documented using a Preferred Reporting Items for Standardization (PRISMA) flow diagram.

#### 3.1.3 Data extraction and quality

Data extraction was conducted independently by two reviewers within the Rayyan platform (14) using a standardized, pilot-tested form to ensure consistency. The extracted variables included demographic characteristics, epidemiological exposure, immune status, clinical presentation, diagnostic methods, therapeutic regimens, and clinical outcomes. Methodological quality was independently assessed by the same two reviewers using the JBI Critical Appraisal Checklist for Case Reports (15). Any disagreements during data extraction or quality appraisal were resolved through discussion and consensus.

#### 3.1.4 Data analysis

Given the rarity of the condition, we performed a descriptive synthesis.

## 4 Results

### 4.1 Selection and characteristics of the study

I The initial database search identified 381 records. After duplicate removal and title/abstract screening, seven reports underwent full-text assessment (16–22). Of these, two were excluded because they did not meet the predefined definition of laboratory-confirmed concurrent coinfection (16, 21), one described remote treated infection/sequelae rather than active coinfection (19), one was excluded because only a conference abstract was available (22), and one could not be assessed because the full text was unavailable (18). Consequently, two studies met the eligibility criteria and were included in the qualitative synthesis alongside the present index case (17, 20) (Figure 6).

**Figure 6:**
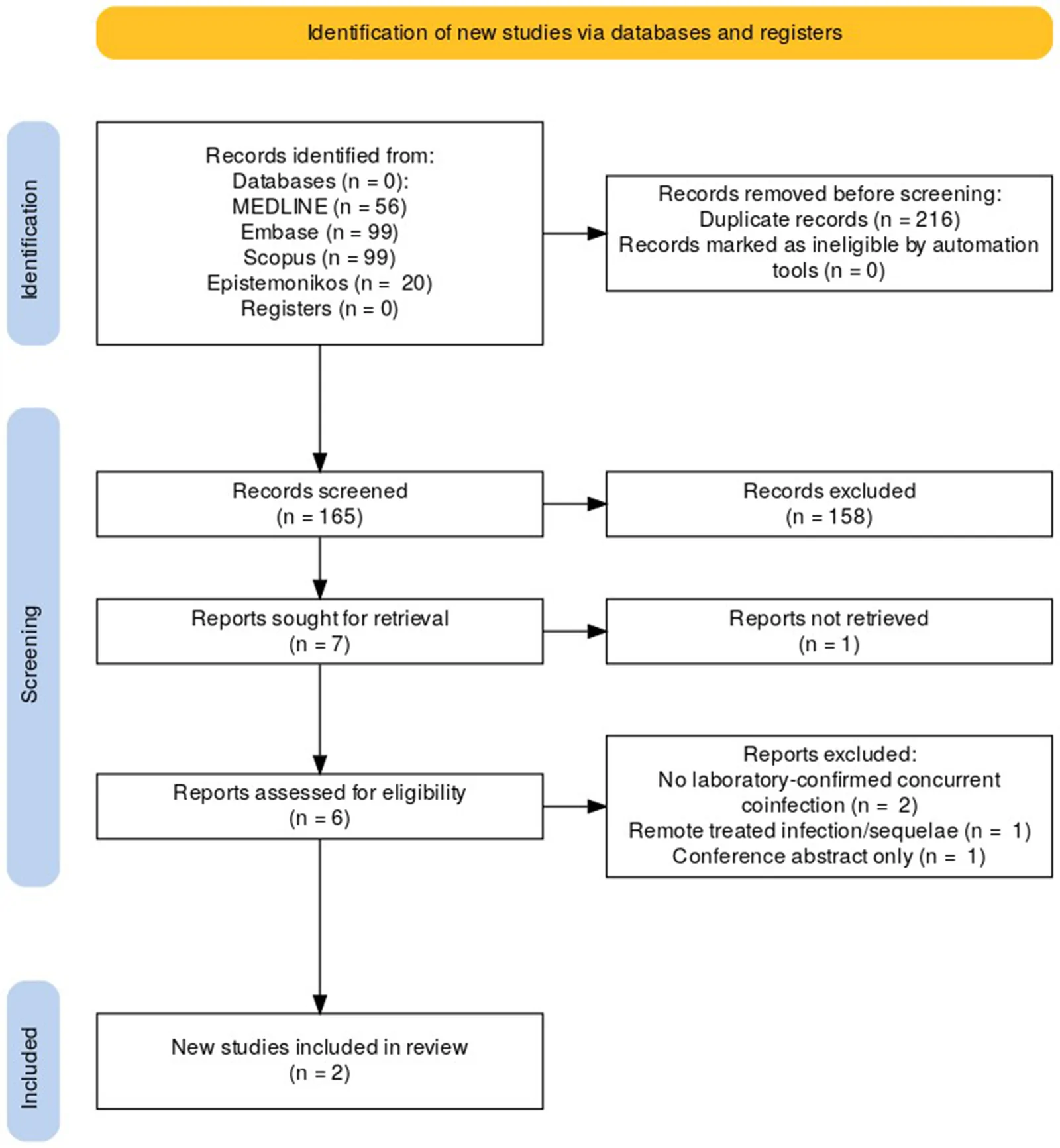
PRISMA 2020 flow diagram.

Most studies adequately reported diagnostic methods and interventions; however, reporting of adverse events was absent across all cases, and temporal sequencing of clinical history was inconsistently described. The results are detailed in Table 2.

**Table 2:** Methodological quality assessment of included studies (JBI Critical Appraisal Checklist for case reports) (15).

Study	Q1	Q2	Q3	Q4	Q5	Q6	Q7	Q8
Salinas et al., 2018 (17)	Y	U	Y	Y	Y	Y	N	Y
Quiroga et al., 2019 (20)	Y	U	Y	Y	Y	Y	N	Y

Y: Yes; N: No; U: Unclear.

Checklist Criteria: Q1. Were patient's demographic characteristics clearly described? Q2. Was the patient's history clearly described and presented as a timeline? Q3. Was the current clinical condition of the patient on presentation clearly described? Q4. Were diagnostic tests or assessment methods and the results described? Q5. Was the intervention(s) or treatment procedure(s) clearly described? Q6. Was the post-intervention clinical condition clearly described? Q7. Were adverse events (harms) or unanticipated events identified and described? Q8. Does the case report provide takeaway lessons?.

### 4.2 Patient characteristics

#### 4.2.1 Demographic characteristics

Among the two published cases of paracoccidioidomycosis and leishmaniasis coinfection, all patients were male older adults. Both cases originated from endemic regions of South America, specifically Brazil and Bolivia. The patients had occupational backgrounds associated with rural and environmental exposure, including farming and lumber work (Table 3).

**Table 3:** Clinical and follow-up characteristics of the reported cases.

Characteristics		Case 1	Case 2
Publication details	Author/year	Salinas et al., 2018 (17)	Quiroga et al., 2019 (20)
Patient characteristics	Age/Sex	74/Male	68/Male
	Geographic origin	Brazil	Bolivia
	Occupation	Lumberjack	Rural worker and enologist
	Comorbidities	Venous insufficiency, alcohol consumption, and smoking	Not reported
	Immunosuppression	No	No
Clinical presentation	Clinical manifestations	Nasal ulcers, rhinorrhea, odynophagia, dysphagia, weight loss	Upper lip and buccal mucosal lesions
	Duration of the illness	2–6 months	4 months
Diagnostic findings	Leishmaniasis diagnosis	Smear, PCR, histopathology	Smear-positive amastigotes
	PCM diagnosis	Serology, PCR, histopathology	Serology, direct examination, histopathology
	Imaging findings	Pulmonary and laryngeal involvement	No imaging abnormalities reported
Treatment and outcomes	Initial treatment	Liposomal amphotericin B (1 mg/kg/day for 30 days), followed by itraconazole (200 mg every 12 h for 12 months)	Liposomal amphotericin B (0.8 mg/kg/day; cumulative dose 2 g), followed by itraconazole (400 mg/day for 6 months)
	Outcome	No recurrence of nasal alveolar lesions at the 6-month follow-up	Lesions resolution at 2 months

#### 4.2.2 Clinical manifestations

The patients were immunocompetent with no evidence of underlying immunosuppression. Relevant comorbidities were reported across cases. The clinical presentation showed substantial variability across cases, encompassing a broad spectrum of disease severity and organ involvement. Mucocutaneous manifestations were a common feature, predominantly affecting nasal and oral regions, with presentations ranging from localized ulcers or scars to more extensive ulcerative and erosive lesions involving multiple anatomical sites, with a subacute to chronic course lasting between 2 and 6 months. Respiratory involvement was observed in more than one case, with imaging findings consistent with chronic PD. Systemic manifestations, such as weight loss and upper aerodigestive symptoms, were reported. Table 3 summarizes the specific clinical manifestations for each case.

#### 4.2.3 Laboratory findings

Laboratory evaluation demonstrated marked variability across cases in the diagnostic methods used for the identification of *Paracoccidioides* spp*.* and *Leishmania* spp*.* Confirmation of coinfection was achieved through different microbiological, histopathological, and serological approaches, reflecting heterogeneity in clinical presentation and diagnostic availability.

*Paracoccidioides* spp*.* infection was established by positive serology, direct microscopy, or histopathological demonstration of characteristic of yeast forms in tissue specimens. In contrast, the diagnosis of leishmaniasis relied on direct examination, polymerase chain reaction, or histopathological findings in mucocutaneous lesions.

Routine laboratory tests and screening for other infectious, malignant, or autoimmune conditions were unremarkable when they were first reported. Comprehensive serological testing and complementary diagnostic studies were performed to exclude alternative diagnoses, supporting the presence of true PCM–leishmaniasis coinfection in all cases.

#### 4.2.4 Treatment and outcomes

Treatment strategies were broadly consistent across cases and were based on established therapeutic approaches for paracoccidioidomycosis and leishmaniasis. All patients received induction therapy with amphotericin B, followed by itraconazole for prolonged oral antifungal treatment. Dosing regimens and treatment duration varied according to clinical severity and organ involvement.

The overall clinical outcomes were favorable. The mucocutaneous lesions were resolved in all reported cases, with no documented relapse during follow-up periods when available.

## 5 Discussion

PCM and leishmaniasis are neglected tropical diseases of major public health relevance in Latin America, with considerable geographic overlap in endemic regions such as Brazil, Colombia, Venezuela, and other Andean countries. This overlap, together with shared occupational and environmental risk factors, particularly among rural populations with sustained exposure to soil and forest ecosystems, supports the biological plausibility of coinfections, although such cases remain rarely reported. Importantly, PCM constitutes a relevant differential diagnosis in patients evaluated for leishmaniasis, especially in mucosal and mucocutaneous forms, as overlapping clinical manifestations and mucocutaneous involvement may lead to diagnostic delays or misclassification, posing a substantial challenge in endemic setting (10).

This report illustrates a case of coinfection with *Paracoccidioides* spp. and *Leishmania* spp. identified as an incidental finding in a male patient originating from an area endemic for *Leishmania* spp. but not formally recognized as endemic for *Paracoccidioides* spp. (5). Nonetheless, this region shares geographic and environmental characteristics with known fungus endemic areas, and the patient's occupational activities are associated with an increased risk of exposure to this microorganism. In this context, the clinical presentation appears to be closely related to the patient's demographic and socioeconomic conditions, compounded by severe PCM, which may have contributed to increased susceptibility to infection and influenced disease progression.

Given that infections caused by these microorganisms share several epidemiological risk factors and pathophysiological characteristics, including alterations in cell-mediated immunity and chronic involvement of mucosal and pulmonary tissues, their coexistence may be underrecognized. However, the limited number of reported cases and the heterogeneity of the available data preclude conclusions regarding causal relationships, temporal sequence, or biological interactions between both infections. Alternative explanations, including shared environmental exposure in endemic areas, diagnostic overlap, delayed recognition of chronic disease, and ascertainment bias, should also be considered. Gonçalves et al. (2020) described a patient with a longstanding history of mucosal leishmaniasis in whom paracoccidioidomycosis was later diagnosed. Although the temporal order of diagnosis was documented, it does not establish the sequence of infection acquisition or a causal relationship between the two diseases. Similar to the other reported cases, this observation highlights the coexistence of both conditions in individuals from endemic settings but does not allow inferences regarding pathogen interactions. The identification and systematic reporting of additional coinfection cases, ideally with detailed clinical, immunological, and microbiological characterization, will be important to determine whether this association reflects shared epidemiological exposures or represents a distinct clinical phenomenon.

A true coinfection was documented in our case by the active presence of both etiological agents in tissue samples, with the identification of *Paracoccidioides* yeast forms and *Leishmania* amastigotes. Although urinary *Histoplasma* antigen testing was positive, this finding is most likely explained by the well-documented cross-reactivity of *Histoplasma* antigen assays with other endemic fungi, including *Paracoccidioides* spp. rather than by concomitant histoplasmosis. Furthermore, the manufacturer of the antigen assay used in this case specifically reports cross-reactivity with *Paracoccidioide*s spp. further supporting this interpretation (Clarus Histoplasma GM EIA, IMMY Inc., Norman, OK, USA) (23, 24). Therefore, the available histopathological and microbiological evidence supported the diagnosis of PCM–leishmaniasis coinfection. From a clinical perspective, however, leishmaniasis likely preceded the development of paracoccidioidomycosis, particularly given that the patient originated from a region endemic for leishmaniasis and presented with features suggestive of chronic infection.

When comparing the present case with the two cases identified in the systematic literature review, all involved older adult men from rural settings or with intense environmental exposure through occupations related to agricultural or forestry activities or frequent contact with soil and plant material. While the previously reported cases originated from regions recognized as endemic for PCM, the index case arose from an area not traditionally considered endemic for PCM. This observation reinforces the role of chronic environmental exposure as a key risk factor for endemic mycoses and leishmaniasis, even in patients without a recognized immunosuppressive disorder, and highlights the need to consider these infections beyond classical endemic settings.

The coexistence of both infections may further contribute to diagnostic delays, particularly in endemic contexts where one disease may obscure the other's clinical recognition. Symptom duration and disease evolution ranged from 2 to 6 months across reported cases, reflecting an insidious and progressive course. This delay is likely exacerbated by socioeconomic factors such as rural living conditions and limited health care access. A potential sequential relationship between infections cannot be excluded, as prior mucosal damage secondary to leishmaniasis may predispose to the subsequent development or clinical expression of paracoccidioidomycosis, complicating diagnosis and delaying appropriate management (25).

A clear predominance of mucocutaneous involvement was observed across the reviewed clinical cases and the index case, with compromise of nasal, oral, and oropharyngeal structures, albeit with marked anatomical heterogeneity. Case 1 presented with nasal lesions and residual scarring; Case 2 showed involvement of the upper lip and buccal mucosa; and the index case exhibited more extensive disease affecting the nasal region, palate, alveolar ridge, and lungs. In the index case, the broader anatomical distribution and associated pulmonary involvement represent a distinctive feature, suggesting a more severe or prolonged clinical spectrum. This heterogeneity in mucocutaneous presentation highlights the substantial clinical overlap between leishmaniasis and paracoccidioidomycosis. In line with these findings, Vicente and Falqueto (2018) (26) demonstrated that specific clinical and anatomical patterns, such as nasal and pharyngeal involvement, are more commonly associated with leishmaniasis, and painful oral lesions and dysphonia with paracoccidioidomycosis may aid in differentiating between these entities. Therefore, the determination of distinguishing clinical, histopathological, and microbiological characteristics for differential diagnosis is critical to improve diagnostic accuracy and optimize patient management, particularly in endemic or resource-limited settings.

The diagnostic approaches used for the identification of *Leishmania* spp. and *Paracoccidioides* spp. were heterogeneous across the reviewed cases, reflecting the complexity of diagnosing these infections when they co-exist. Diagnosis relied on combinations of clinical evaluation, histopathology, direct microscopic examination, serology, molecular techniques, and imaging studies, although availability and performance varied among cases. Current guidelines for both PCM and leishmaniasis recommend integrating epidemiological and clinical findings with laboratory confirmation. For PCM, direct visualization of the characteristic yeast forms in clinical specimens, histopathological examination, and microbiological culture remain the cornerstone of diagnosis. For leishmaniasis, demonstration of amastigotes in tissue samples through histopathological or parasitological examination is recommended, with serological methods such as indirect immunofluorescence (IFA) serving as complementary diagnostic tools (10, 27). This variability represented a relevant limitation in the index case, as definitive identification required multiple complementary studies and extensive exclusion of alternative diagnoses (28, 29). The lack of institutional availability of molecular diagnostic tools, such as polymerase chain reaction (PCR)-based assays, precluded precise species-level identification of both pathogens. This limitation is especially relevant given the importance of molecular characterization for diagnostic confirmation and epidemiological understanding, even though species-level identification is infrequently reported and systematic surveillance remains limited.

Furthermore, the limited number of reports in the literature highlights the diagnostic challenges associated with the clinical and epidemiological overlap of these entities. This situation underscores the importance of maintaining a high index of clinical suspicion and considering a broad range of differential diagnoses in patients from endemic areas, even in the absence of evident immunosuppression, which is essential for timely diagnosis and appropriate management.

An additional relevant limitation of this report is the limited clarity regarding the current epidemiology of PCM, particularly in Colombia. Although clinical case reports and more recent descriptions from other Latin American countries up to 2025 have been published in the national literature between 2000 and 2018, the true prevalence of the disease and its epidemiological behavior remain poorly defined (7). This contrasts with conditions such as leishmaniasis, for which more robust and up-to-date epidemiological data are available.

This disparity may be partially explained by the mandatory notification of leishmaniasis cases to territorial health authorities in Colombia, a requirement that currently does not apply to PCM. In this context, there is a clear need to strengthen active case finding and improve epidemiological surveillance and reporting systems for PCM, given its relevance to public health not only at the national level but also across Latin America, where the disease is associated with a substantial burden of morbidity and complications, even after adequate medical treatment (2).

Finally, systematic identification and epidemiological follow-up of cases of both infections individually and in coinfection scenarios could contribute to a better understanding of potential epidemiological shifts of these microorganisms, thereby enabling the implementation of more effective preventive strategies for at-risk populations. This is particularly relevant in Latin America's socioeconomic context, where agriculture represents a major and growing economic sector, as reflected in recent gross domestic product (GDP) indicators. However, because only two published case reports met the eligibility criteria, the findings of the present review should be interpreted as descriptive evidence and should not be used to infer consistent epidemiological, clinical, or therapeutic patterns. Additional reports and prospective studies are needed to better characterize this uncommon coinfection.

## Data Availability

The original contributions presented in the study are included in the article/Supplementary Material, further inquiries can be directed to the corresponding author/s.

## References

[1] Sanabria Peña CL Alarcón Tarazona ML Alarcón IE Jaimes Daza MF Sanabria Peña CL Alarcón Tarazona ML. Paracoccidioidomicosis. Una enfermedad multisistémica. Acta Med Colomb. (2018) 43(2):111–4.

[2] Venturini J Fernandez NB De Macedo PM Cavalcante RS Caceres DH Borges CL. Paracoccidioidomycosis in the 21st century: challenges and milestones. PLoS Negl Trop Dis. (2026) 20(1):e0013819. 10.1371/journal.pntd.0013819 PubMed PMID: 41494000; PubMed Central PMCID: PMC12774349. 41494000 PMC12774349

[3] Fernández NB Toranzo A Farias L Canteros CE. Diagnóstico micológico de paracoccidioidomicosis en un hospital de área no endémica: metodología clásica y molecular. Biomédica. (2023) 43(Sp. 1):132–43. 10.7705/biomedica.6888 PMC10569774 37721907

[4] Flórez CJM Castro NC. Paracoccidioidomicosis. Medicina UPB. (2015) 34(2):126–37. 10.18566/medupb.v34n2.a05

[5] Torrado E Castañeda E De La Hoz F Restrepo A. Definition of paracoccidioidomycosis endemic areas in Colombia. Biomédica. (2000) 20(4):327–34. 10.7705/biomedica.v20i4.1076

[6] Rodrigues AM Hagen F Puccia R Hahn RC De Camargo ZP. Paracoccidioides and paracoccidioidomycosis in the 21st century. Mycopathologia. (2023) 188(1–2):129–33. 10.1007/s11046-022-00704-y 36633737

[7] Arias Ramos D Alzate JA Giraldo Montoya ÁM Trujillo YA Arias Ramos LY. Thinking in paracoccidioidomycosis: a delayed diagnosis of a neglected tropical disease, case report and review of clinical reports and eco-epidemiologic data from Colombia since the 2000. BMC Infect Dis. (2020) 20(1):119. 10.1186/s12879-020-4864-8 32041552 PMC7011283

[8] Abadías-Granado I Diago A Cerro PA Palma-Ruiz AM Gilaberte Y. Leishmaniasis cutánea y mucocutánea. Actas Dermo-Sifiliográficas. (2021) 112(7):601–18. 10.1016/j.ad.2021.02.008 33652011

[9] Pan American Health Organization. Leishmaniases: Epidemiological Report of the Americas [Internet]. Report No. 7. Washington, DC: PAHO (2019). Available online at: https://www.who.int/publications/i/item/PAHO-leish-Epidemio-Report-Americas-7-2019 (Accessed April 13, 2026).

[10] Ministerio de Salud y Protección Social. Lineamientos de Atención clínica Integral para Leishmaniasis en Colombia. Versión 4 [Internet]. Bogotá (Colombia): Ministerio de Salud y Protección Social (2023). Available online at: https://www.minsalud.gov.co/sites/rid/Lists/BibliotecaDigital/RIDE/VS/PP/PAI/Lineamientos-leishmaniasis.pdf (Accessed July 27, 2026).

[11] Instituto Nacional De Salud. Leishmaniasis en Colombia, Semana Epidemiológica 24 del 2025. [Boletín Epidemiológico] [Internet]. Colombia. (2025). Available online at: https://www.ins.gov.co/BibliotecaDigital/2025-boletin-epidemiologico-semana-26.pdf (Accessed April 13, 2026).

[12] Aromataris E Lockwood C Porritt K Pilla B Jordan Z, editors. JBI Manual for Evidence Synthesis [Internet]. Adelaide: JBI (2024). 10.46658/JBIMES-24-01

[13] Page MJ McKenzie JE Bossuyt PM Boutron I Hoffmann TC Mulrow CD. The PRISMA 2020 statement: an Accessed guideline for reporting systematic reviews [Internet] (2021). p. n71. 10.1136/bmj.n71 PMC8005924 33782057

[14] Ouzzani M Hammady H Fedorowicz Z Elmagarmid A. Rayyan-a web and mobile app for systematic reviews. Syst Rev. (2016) 5(1):210. 10.1186/s13643-016-0384-4 PubMed PMID: 27919275; PubMed Central PMCID: PMC5139140. 27919275 PMC5139140

[15] Moola S Munn Z Tufanaru C Aromataris E Sears K Sfetcu R. Systematic reviews of etiology and risk (2020). In: Aromataris E Lockwood C Porritt K Pilla B Jordan Z, editors. JBI Manual for Evidence Synthesis. (2024). Available from: https://synthesismanual.jbi.global (Accessed April 13, 2026).

[16] De Jesus Oliveira Gonçalves CA Carneiro JT De Souza Cruz EL De Sousa Neves Filho F Rivadeneira Cárdenas RC Guimarães DM. Parasitological association between human leishmaniosis mucosa and paracoccidioidomycosis. Case report. Int J Surg Case Rep. (2020) 76(C):170–3. 10.1016/j.ijscr.2020.09.128 PMC7553885 33035956

[17] Salinas ME Sehtman A Roch V Allevato MA de los Juárez MA. Leishmaniasis mucocutánea asociada a infección diseminada por paracoccidioides brasiliensis. Prensa méd Argent. (2018) 104(2):59–63. Located at: LILACS. Available online at: https://prensamedica.com.ar/LPMA_V104_N02_comp.pdf (Accessed April 13, 2026).

[18] Encinas Maldonado S Garabito Rollano F. Comorbilidad: paracoccidiodomicosis y leishmaniasis cutánea caso clinico revisión de literatura. Actualización Médico Científica. (2011) 6(6):36–41. Located at: LIBOCS. Available online at: https://pesquisa.bvsalud.org/portal/resource/es/biblio-1585280 (Accessed April 13, 2026).

[19] Leffa Rosa CA Begnini GJ Tychanowicz Kolodziejwski W Da Silva P Valesti Oliveira C De Araujo MR. Functional reestablishment in the hospital setting of a patient with sequelae from leishmaniasis and paracoccidioidomy. Oral Surg Oral Med Oral Pathol Oral Radiol. (2024) 137(6):e171. 10.1016/j.oooo.2023.12.117

[20] Quiroga CF Candiz ME Mazzaroni S Olivares L Maronna E. Paracoccidioidomicosis asociada a leishmaniasis. Dermatología Argentina. (2019) 25(3):125–7. Available online at: https://dermatolarg.org.ar/index.php/dermatolarg/es/article/view/1888 (Accessed April 14, 2026).

[21] Vieira TS Reis TMG Meira SS. Diagnóstico diferencial de paracoccidioidomicose e leishmaniose tegumentar cutânea: relato de caso. Saúde.com. (2016) 12(3):675–8. Available online at: https://periodicos2.uesb.br/rsc/article/view/429 (Accessed April 14, 2026).

[22] Tudanca V Sierra M Cornet M Paravano L Fernandez N Farias L. Coinfección leishmaniasis y paracoccidioidomicosis: a propósito de un caso. In: PE281. Argentina (2017). Available online at: https://infectologia.info/abstracts/coinfeccion-leishmaniasis-y-paracoccidioidomicosis-a-proposito-de-un-caso/ (Accessed April 20, 2026).

[23] Guimarães AJ Nosanchuk JD Zancopé-Oliveira RM. Diagnosis of histoplasmosis. Braz J Microbiol. (2006) 37(1):1–13. 10.1590/S1517-83822006000100001. PubMed PMID: 20445761; PubMed Central PMCID: PMC2863343. PMC2863343 20445761

[24] Azar MM Hage CA. Laboratory diagnostics for histoplasmosis. J Clin Microbiol. (2017) 55(6):1612–20. 10.1128/JCM.02430-16 PubMed PMID: 28275076; PubMed Central PMCID: PMC5442517. 28275076 PMC5442517

[25] Ibáñez Franco EJ González Báez CA Aldama Caballero ABF Penayo Aguilera TDJ Montoya Bueno C Pereira Brunelli JG. Paracoccidioidomicosis en paciente con secuelas de leishmaniasis en mucosas. Revista Virtual de la Sociedad Paraguaya de Medicina Interna. (2024) 11(1):56. 10.18004/rvspmi/2312-3893/2024.e11142408

[26] Vicente CR Falqueto A. Differentiation of mucosal lesions in mucocutaneous leishmaniasis and paracoccidioidomycosis. PLoS One. (2018) 13(11):e0208208. 10.1371/journal.pone.0208208. PubMed PMID: 30475920; PubMed Central PMCID: PMC6258372. 30475920 PMC6258372

[27] Micologiacolombia Paracoccidioidomicosis [Internet]. Available online at: https://micologiacolombia.ins.gov.co/Paginas/Paracoccidioidomicosis/Paracoccidioidomicosis-todos.aspx (Accessed July 27, 2026).

[28] Mathison BA Bradley BT. Review of the clinical presentation, pathology, diagnosis, and treatment of leishmaniasis. Lab Med. (2023) 54(4):363–71. 10.1093/labmed/lmac134 36468667

[29] Peçanha PM Peçanha-Pietrobom PM Grão-Velloso TR Rosa Júnior M Falqueto A Gonçalves SS. Paracoccidioidomycosis: what we know and what is new in epidemiology, diagnosis, and treatment. J Fungi (Basel. (2022) 8(10):1098. 10.3390/jof8101098 PubMed PMID: 36294662; PubMed Central PMCID: PMC9605487. PMC9605487 36294662

