# Supplementary material for: Coinfection of paracoccidioidomycosis and mucocutaneous leishmaniasis: a case report and systematic review
Source: Front Med (Lausanne). 2026 Oct 8;13:1892049. doi: 10.3389/fmed.2026.1892049 (PMC13686874; doi:10.3389/fmed.2026.1892049)
Supplement: Supplementary file 1 [file Table1.docx]

**SUPPLEMENTARY TABLE S1. Complete Search Strategy**

**Ovid MEDLINE <1946 to April 14, 2026>**

| **No.** | **Search strategy** | **Results** |
| --- | --- | --- |
| 1 | exp Paracoccidioidomycosis/ | 2070 |
| 2 | Paracoccidioides/ | 1705 |
| 3 | (paracoccidioidomycosis or Paracoccidioides).ti,ab. | 2996 |
| 4 | 1 or 2 or 3 | 3315 |
| 5 | exp Leishmaniasis/ | 27524 |
| 6 | Leishmania/ | 9245 |
| 7 | (leishmaniasis or Leishmania).ti,ab. | 42425 |
| 8 | 5 or 6 or 7 | 45620 |
| 9 | 4 and 8 | 70 |
| 10 | limit 9 to humans | 56 |

**Embase sessions results (14 April 2026)**

| **No.** | **Search strategy** | **Results** |
| --- | --- | --- |
| #10 | #4 AND #8 AND [humans]/lim | 99 |
| #9 | #4 AND #8 | 121 |
| #8 | #5 OR #6 OR #7 | 61221 |
| #7 | leishmaniasis:ab,kw,ti OR leishmania:ab,kw,ti | 50171 |
| #6 | 'leishmania' | 43186 |
| #5 | 'leishmaniasis' | 46631 |
| #4 | #1 OR #2 OR #3 | 3823 |
| #3 | 'paracoccidioides' | 3034 |
| #2 | paracoccidioidomycosis:ab,kw,ti OR paracoccidioides:ab,kw,ti | 3438 |
| #1 | exp AND ('paracoccidioidomycosis'/exp OR paracoccidioidomycosis) | 33 |

**Scopus Search (14 April 2026)**

(TITLE-ABS-KEY ("Paracoccidioidomycosis" OR "Paracoccidioides")

AND TITLE-ABS-KEY ("Leishmaniasis" OR "Leishmania"))

AND (LIMIT-TO (EXACTKEYWORD, "Human"))

**Results:** 99

**LILACS (14 April 2026)**

| **Search strategy** | **Results** |
| --- | --- |
| ("Paracoccidioidomycosis" OR "Paracoccidioides") | 4054 |
| ("Leishmaniasis" OR "Leishmania") | 18773 |
| ("Paracoccidioidomycosis" OR "Paracoccidioides") AND ("Leishmaniasis" OR "Leishmania") | 103 |

**Epistemonikos (14 April 2026)**

(title:((title:("Paracoccidioidomycosis" OR "Paracoccidioides")

OR abstract:("Paracoccidioidomycosis" OR "Paracoccidioides"))

AND (title:("Leishmaniasis" OR "Leishmania")

OR abstract:("Leishmaniasis" OR "Leishmania")))

OR abstract:((title:("Paracoccidioidomycosis" OR "Paracoccidioides")

OR abstract:("Paracoccidioidomycosis" OR "Paracoccidioides"))

AND (title:("Leishmaniasis" OR "Leishmania")

OR abstract:("Leishmaniasis" OR "Leishmania"))))

**Results:** 20

**SUPPLEMENTARY TABLE S2 Eligible reports and final study selection decisions**

| **Reference** | **Title** | **Final Decision** | **Reason** |
| --- | --- | --- | --- |
| de Jesus Oliveira Gonçalves et al, 2020 [16] | Parasitological association between human leishmaniosis mucosa and paracoccidioidomycosis. Case report | Excluded | No laboratory-confirmed coinfection. Diagnosis of leishmaniasis was based on the Montenegro skin test rather than microbiological, histopathological, or molecular confirmation. |
| Salinas et al, 2018 [17] | Leishmaniasis mucocutánea asociada a infección diseminada por *Paracoccidioides brasiliensis* | Included |  |
| Encinas Maldonado et al, 2011 [18] | Comorbilidad: paracoccidiodomicosis y leishmaniasis cutánea caso clínico revisión de literatura | Not retrieved | Full text unavailable |
| Leffa Rosa et al, 2024 [19] | Functional reestablishment in the hospital setting of a patient with sequelae from leishmaniasis and paracoccidioidomy | Excluded | Remote treated infection/sequelae.  The report describes sequelae of previous paracoccidioidomycosis and leishmaniasis rather than concurrent active coinfection. |
| Quiroga et al, 2019[20] | Paracoccidioidomicosis asociada a leishmaniasis | Included | - |
| Vieira et al, 2016. [21] | Diagnóstico diferencial de paracoccidioidomicose e leishmaniose tegumentar cutânea: relato de caso | Excluded | No laboratory-confirmed concurrent coinfection |
| Tudanca et al, 2017. [22] | Coinfección leishmaniasis y paracoccidioidomicosis: a propósito de un caso | Excluded | Conference abstract (poster); not eligible for inclusion because full study details were unavailable. |
