# Supplementary material for: Coinfection of paracoccidioidomycosis and mucocutaneous leishmaniasis: a case report and systematic review
Source: Front Med (Lausanne). 2026 Oct 8;13:1892049. doi: 10.3389/fmed.2026.1892049 (PMC13686874; doi:10.3389/fmed.2026.1892049)
Supplement: Supplementary file 2 [file Table2.docx]

**SUPPLEMENTARY TABLE S2 Eligible reports and final study selection decisions**

| **Reference** | **Title** | **Final Decision** | **Reason** |
| --- | --- | --- | --- |
| de Jesus Oliveira Gonçalves et al, 2020 [16] | Parasitological association between human leishmaniosis mucosa and paracoccidioidomycosis. Case report | Excluded | No laboratory-confirmed coinfection. Diagnosis of leishmaniasis was based on the Montenegro skin test rather than microbiological, histopathological, or molecular confirmation. |
| Salinas et al, 2018 [17] | Leishmaniasis mucocutánea asociada a infección diseminada por *Paracoccidioides brasiliensis* | Included |  |
| Encinas Maldonado et al, 2011 [18] | Comorbilidad: paracoccidiodomicosis y leishmaniasis cutánea caso clínico revisión de literatura | Not retrieved | Full text unavailable |
| Leffa Rosa et al, 2024 [19] | Functional reestablishment in the hospital setting of a patient with sequelae from leishmaniasis and paracoccidioidomy | Excluded | Remote treated infection/sequelae.  The report describes sequelae of previous paracoccidioidomycosis and leishmaniasis rather than concurrent active coinfection. |
| Quiroga et al, 2019[20] | Paracoccidioidomicosis asociada a leishmaniasis | Included | - |
| Vieira et al, 2016. [21] | Diagnóstico diferencial de paracoccidioidomicose e leishmaniose tegumentar cutânea: relato de caso | Excluded | No laboratory-confirmed concurrent coinfection |
| Tudanca et al, 2017. [22] | Coinfección leishmaniasis y paracoccidioidomicosis: a propósito de un caso | Excluded | Conference abstract (poster); not eligible for inclusion because full study details were unavailable. |
